# Diagnoses indicating pain and analgesic drug prescription in patients with dementia: a comparison to age- and sex-matched controls

**DOI:** 10.1186/1471-2318-14-20

**Published:** 2014-02-12

**Authors:** Falk Hoffmann, Hendrik van den Bussche, Birgitt Wiese, Gerd Glaeske, Hanna Kaduszkiewicz

**Affiliations:** 1 Centre for Social Policy Research, Division Health Economics, Health Policy and Outcomes Research, University of Bremen, Postfach 33 04 40, D-28334 Bremen, Germany; 2 Institute of Primary Medical Care, University Medical Center Hamburg-Eppendorf, Hamburg, Germany; 3 Institute of Biometrics, Hannover Medical School, Hannover, Germany

**Keywords:** Dementia, Comorbidity, Health services research, Pain, Analgesics

## Abstract

**Background:**

The evidence of undertreatment of pain in patients with dementia is inconsistent. This may largely be due to methodological differences and shortcomings of studies. In a large cohort of patients with incident dementia and age- and sex-matched controls we examined (1) how often they receive diagnoses indicating pain, (2) how often they receive analgesics and (3) in which agents and formulations.

**Methods:**

Using health insurance claims data we identified 1,848 patients with a first diagnosis of dementia aged ≥ 65 years and 7,385 age- and sex-matched controls. We analysed differences in diagnoses indicating pain and analgesic drugs prescribed between these two groups within the incidence year. We further fitted logistic regression models and stepwise adjusted for several covariates to study the relation between dementia and analgesics.

**Results:**

On average, patients were 78.7 years old (48% female). The proportions receiving at least one diagnosis indicating pain were similar between the dementia and control group (74.4% vs. 72.5%; p = 0.11). The proportion who received analgesics was higher in patients with dementia in the crude analysis (47.5% vs. 44.7%; OR: 1.12; 95% CI: 1.01-1.24), but was significantly lower when adjusted for socio-demographic variables, care dependency, comorbidities and diagnoses indicating pain (OR: 0.78; 95% CI: 0.68-0.88). Analgesics in liquid form such as metamizole and tramadol were more often used in dementia.

**Conclusions:**

Our findings show a comparable documentation of diagnoses indicating pain in persons with incident dementia compared to those without. However, there still seems to be an undertreatment of pain in patients with dementia. Irrespective of dementia, analgesics seem to be more often prescribed to sicker patients and to control pain in the context of mobility.

## Background

Pain is a common symptom in older persons. Findings from clinical and experimental studies show that pain in patients with dementia is as frequent and intense as in patients without dementia, even if less reported [1]. Dementia guidelines often mention pain as a possible cause of ‘Behavioral and Psychological Symptoms of Dementia’ (BPSD) [2,3]. A recently published study showed that an appropriate treatment of pain in nursing home residents may reduce the incidence of agitation and neuropsychiatric symptoms [4].

However, there is evidence that pain in dementia may remain undetected due to difficulties in communication [5-8]. Along with problems of detection, there is inconsistent evidence of undertreatment of pain in patients with dementia. Some studies found a lower proportion of painkillers prescribed for dementia patients [8-13], others show no differences for (strong) pain medication [14-16] or even more frequent use in cognitively impaired patients [17]. Haasum et al. show higher use of paracetamol (acetaminophen) in persons with dementia and no differences in the use of any analgesics, opioids and NSAIDs after adjustment for confounders. They interpret their findings as a possible reflection of an ‘increased awareness of pain and pain management in persons with dementia’ in comparison to older studies [17]. A recent Finnish study in turn showed partially different results. There, dementia patients were given fewer opioids, but more often strong opioids (especially fentanyl) [18].

The diverging results of the cited studies may largely be due to methodological differences and shortcomings. The two more recent Scandinavian studies were population based with a high number of included patients [17,18]. All the other studies refer to much smaller, mostly nursing home based populations [8-16]. Most studies investigated the use of only some painkiller groups (e.g. only opioids) and all studies included prevalent dementia patients, i.e. dementia patients in very different stages of the disease.

The aim of this study was to investigate in a large cohort of newly diagnosed dementia patients compared to age- and sex-matched controls

• the occurrence of pain (and types of diagnoses indicating pain),

• the prescribing of analgesics (and which agents and formulations) and

• factors associated with the prescription of analgesics within the incidence year.

## Methods

### Design and study population

For this study, we used pseudonymized claims data of the Gmünder ErsatzKasse (GEK), a statutory health insurance company which insured 1.7 million people located in all regions of Germany during the study period (2% of the German population). The data used in this study are not publicly available. Patients with a first diagnosis of dementia in ambulatory medical care between the first quarter of the year 2005 and the first quarter of 2006 were identified. Quarters had to be chosen because they form the basic time period for coding diagnoses in ambulatory care in Germany and only the quarter in which a diagnosis occurred is available in claims data. Patients with incident dementia were included if the following criteria were fulfilled:

• age of at least 65 years,

• at least one ICD-10 code for dementia from the following list (F00.x, F01.x, F02.0, F02.3, F03, G30.x, G31.0, G31.1, G31.82, G31.9, and R54) in ambulatory medical care in at least 3 of 4 consecutive quarters,

• at least 4 quarters free from this diagnoses before and

• continuous insurance period of four quarters before and after the first code was recorded.

Furthermore, we matched up to four controls for each patient with incident dementia using the nearest neighbour matching method of the matchit package of the statistical software ‘R’. Matching criteria were sex, year of birth, amount of contacts with physicians, and the number of consulted physicians four quarters before incidence. Thus patients with and without dementia are comparable with regard to ambulatory health care utilisation. Further requirements for the control group were the absence of dementia diagnoses and a continuous insurance period within the 2 years of observation. The quarter in which one of the codes indicating dementia appears for the first time is called the ‘incidence quarter’. The incidence quarter of the controls was that of the case. This and the following 3 quarters are referred to as the ‘incidence year’, forming the study period for all of our following analyses.

The selection of patients and controls has been described in more detail elsewhere [19-21].

### Diagnoses indicating pain, analgesic prescribing and covariates

The International Classification of Diseases (ICD-10) lists only few specific pain diagnoses. Thus, relying only on these diagnoses for the identification of patients with pain in claims data would probably underestimate its occurrence. Freytag et al. used classification and regression trees (CART) for the identification of patients with pain based on health insurance claims data [22]. They developed 9 types of pain to which 77.1% of all patients with at least two opioid prescriptions within a one year period could be allocated. By aggregating these 9 pain types and further including unspecific diagnoses, nearly all persons with at least one prescription of tramadol (91.7%) could be identified as patients with pain in a recent analysis [23]. Based on these studies we used the following pain types and ICD-10 diagnoses:

• Cancer pain (ICD-10: C20, C34, C64, C77-C80, C85, C90, Z51),

• Pain due to arthritis or osteoarthritis (ICD-10: M06, M13, M15-M19, M25),

• Pain due to fractures (ICD-10: S12, S22, S32, S42, T02, T08, T91),

• Pain due to multimorbidity and care dependency (ICD-10: L89, L97, L98),

• Headache (ICD-10: G43, G44, R51),

• Neuropathic pain (ICD-10: G50, G54, G56-G58, G62, G63, M79, M89),

• Back pain (ICD-10: M43, M45-M51, M53, M54, M81, M82) and

• Pain, not elsewhere classified (ICD-10: R52).

At least one of these diagnoses indicating pain had to be documented in ambulatory medical care within the incidence year, or the respective year in controls.

The prescription of drugs within the German statutory health insurances is allowed for all physicians (including general practitioners as well as specialists) working in the ambulatory sector. The hospital sector does not play a notable role in the prescription of drugs. Prescriptions relate to both community-dwelling persons and those living in nursing homes. With some exceptions including over-the-counter (OTC) medications, prescribed drugs are reimbursed by the statutory insurances requiring a co-payment of 10% of the price with a minimum of 5 € and a maximum of 10 € for each prescription. However, co-payment exemptions are available for the chronically ill.

We identified all drugs according to the so-called analgesic ladder of the World Health Organization (WHO). However, acetylsalicylic acid (ASA) and paracetamol were not included in the analyses because both drugs are not reimbursed by the statutory health insurances and are solely bought OTC in Germany. We also studied the route of administration with a special focus on liquid oral and transdermal formulations.

We used two measures of comorbidity: (1) levels of care dependency and (2) the number of prescribed medications. Services from the German long term care insurance are provided to those who require support in their activities of daily living including personal hygiene, eating, mobility and – separate from personal care – housekeeping. There are three levels of care dependency related to the estimated time required for assistance indicating moderate (level I), severe (level II) and severest care dependency (level III) [24,25]. If care dependency changed within the incidence year we included the highest level in our analyses. Furthermore, it has been shown that the number of distinct medications prescribed performed well as a predictor for health services utilization and mortality in older persons [26,27]. This easily applicable measure of degree of comorbidity is often used in studies based on claims data [28,29]. For our analyses, we categorised the number of prescribed medications within the incidence year into quartiles (including all prescriptions filled in pharmacies).

### Statistical analysis

Our main focus lies on differences between patients with and without dementia. After a descriptive characterisation of the study cohort, the proportion of patients with at least one diagnosis indicating pain in the incidence year of dementia or the respective year in those without dementia was estimated. We further compared the agents prescribed to the two groups within the incidence year. The proportions of patients prescribed analgesics were compared on the basis of at least one prescription per patient. We grouped analgesics according to the WHO analgesic ladder as well as to the route of administration. Differences in proportions between the dementia and control group were analysed with the chi-square test.

To study the relation between the prescription of at least one analgesic drug and incident dementia, we fitted logistic regression models and stepwise adjusted for several covariates. First, an univariate analysis was performed (model 1). In model 2, we adjusted for demographic variables and care dependency including age (65–74, 75–84, 85+ years); sex (male, female); level of care dependency (4 categories) and place of residence (nursing home, community-dwelling). For model 3, further comorbidity measures and diagnoses indicating pain were entered in the multivariate model: the quartiles of the numbers of prescribed medications (4 categories) as a proxy for the severity of comorbidity as well as the 8 different pain types (yes, no). Crude and adjusted odds ratios (OR) with 95% confidence intervals (95% CI) were estimated.

We performed all statistical analyses with SAS for Windows version 9.2 (SAS Institute Inc., Cary, NC).

The study was conducted according to the principles expressed in the Declaration of Helsinki. The use of health insurance claims data for scientific research is regulated by the German Code of Social Law (SGB X) and no informed consent has to be obtained. According to the Good Practice of Secondary Data Analysis, a national guideline for the use of administrative databases, no approval of an ethical committee is required [30].

## Results

### Characteristics of the study cohort

Baseline characteristics of the 1,848 patients with dementia and 7,385 controls are shown in Table 1. Because of the matching, sex and age are similar in both cohorts, but there are noticeable differences regarding care dependency. Almost half of the dementia patients (44.4%) are assigned to one of the three care levels within the incidence year, whereas this proportion is only 12.9% for the control group. Patients with dementia received on average more different medications than controls (9.7 vs. 7.6).

**Table 1:** Baseline characteristics of patients with incident dementia and matched controls

**Baseline characteristics**	**Dementia group (n = 1,848)**	**Control group (n = 7,385)**
Mean age, in years (SD)	78.7 (7.4)	78.7 (7.4)
Age groups, in years		
65-74	30.6%	30.6%
75-84	47.1%	47.3%
85+	22.3%	22.1%
Sex		
Male	52.4%	52.5%
Female	47.6%	47.5%
Level of long term care		
None	55.6%	87.1%
I	20.7%	7.6%
II	18.5%	4.4%
III	5.3%	0.9%
Place of residence		
Nursing home	20.1%	3.2%
Community-dwelling	79.9%	96.8%
Number of prescribed medications; mean (SD)	9.7 (5.7)	7.6 (5.4)
25th percentile (Q1)	6	4
50th percentile (Q2)	9	7
75th percentile (Q3)	13	11

Although there are pain types from which patients with dementia suffer more often than controls (for instance, pain due to fractures and pain due to multimorbidity and care dependency), the proportion of patients receiving at least one diagnosis indicating pain is similar in both groups (74.4% vs. 72.5%; p = 0.11) (Table 2).

**Table 2:** Types of pain in patients with incident dementia and matched controls

**Type of pain**	**Dementia group (n = 1,848) (in %)**	**Control group (n = 7,385) (in %)**	**p-value**
Back pain	51.7	53.2	0.23
Pain due to arthritis or osteoarthritis	41.2	41.8	0.64
Neuropathic pain	19.4	16.5	0.0037
Pain due to fractures	4.6	3.0	0.0006
Pain due to multimorbidity and care dependency	9.8	4.0	<0.0001
Pain, not elsewhere classified	9.0	6.0	<0.0001
Headache	6.3	5.0	0.0331
Cancer pain	5.0	5.9	0.14
Total (at least one pain type)	74.4	72.5	0.11

### Prescribing of analgesic drugs

A total of 18,868 dispensings of analgesic drugs were included in the analyses. The top ten most frequently used substances in the dementia and control group are shown in Table 3. Noticeably, the top five substances already make up more than three quarters of the prescriptions in both groups. Metamizole (27.4% vs. 17.8%) and tramadol (13.5% vs. 10.2%) accounted for a higher proportion of prescriptions in patients with dementia while diclofenac (mono) (18.9% vs. 26.3%) and tilidine/naloxone (6.0% vs. 7.9%) were more often prescribed for controls (p < 0.0001 for all comparisons).

**Table 3:** Top ten most prescribed analgesic drugs for patients with incident dementia and matched controls

**Dementia group (n = 1,848; 4,441 prescriptions)**		**Control group (n = 7,385; 14,427 prescriptions)**	
**Substance**	**Proportion (in %)**	**Substance**	**Proportion (in %)**
Metamizole (dipyrone)	27.4	Diclofenac (mono)	26.3
Diclofenac (mono)	18.9	Metamizole (dipyrone)	17.8
Tramadol	13.5	Ibuprofen	13.3
Ibuprofen	12.5	Tramadol	10.2
Fentanyl	6.7	Tilidine/naloxone	7.9
Tilidine/naloxone	6.0	Fentanyl	6.3
Oxycodone	1.8	Oxycodone	2.1
Codeine/paracetamol	1.8	Codeine/paracetamol	2.1
Buprenorphine	1.7	Morphine	2.0
Flupirtine	1.6	Buprenorphine	1.7

The proportion of patients with at least one analgesic drug prescription during the incidence year was significantly higher in patients with dementia compared to controls (47.5% vs. 44.7%; p = 0.03). This difference is mainly due to a higher prescription prevalence with WHO step II opioids and liquid oral substances in the dementia group (Table 4). Again, more patients with dementia received metamizole (18.7% vs. 12.8%) and tramadol (8.8% vs. 6.0%). Both agents are available in liquid oral formulations in Germany. Also, significantly more patients with dementia got prescriptions for transdermal opioids, but the overall prevalence is relatively low.

**Table 4:** Proportion of patients receiving a prescription for at least one analgesic drug according to WHO ladder and route of administration for patients with incident dementia and matched controls

**Characteristics**	**Dementia group (n = 1,848) (in %)**	**Control group (n = 7,385) (in %)**	**p-value**
WHO step I drugs			
Diclofenac (mono)	19.5	21.6	0.04
Metamizole	18.7	12.8	<0.0001
Total	42.8	40.7	0.11
WHO step II drugs			
Tramadol	8.8	6.0	<0.0001
Tilidine/naloxone	4.1	3.9	0.77
Total	14.7	11.4	0.0001
WHO step III drugs			
Fentanyl	2.5	2.0	0.16
Oxycodone	0.8	0.9	0.65
Total	4.2	3.6	0.19
Route of administration			
Liquid oral	19.2	12.8	<0.0001
Transdermal	3.1	2.3	0.03
Total (at least one analgesic)	47.5	44.7	0.03

### Factors associated with prescribing

Results of the univariate and multivariate logistic regression analyses are presented in Table 5. As reported above, significantly more patients with dementia received prescriptions of analgesics in the crude analysis. However, this association was no longer found when we adjusted for age, sex, level of care and place of residence in model 2 (OR: 0.96; 95% CI: 0.86-1.07). After including the number of prescribed medications as a comorbidity measure as well as diagnoses indicating pain (model 3), we found that analgesics were significantly less often prescribed for patients with dementia (OR: 0.78; 95% CI: 0.68-0.88). Female sex and long-term care level II (but not level III) were associated with prescriptions of analgesics in both models and the number of prescribed medications showed a strong impact whereas place of residence had no influence. Within the types of pain investigated, pain due to osteoarthritis, fractures, neuropathic pain and back pain showed statistically significant associations with prescriptions of analgesics in the multivariate model.

**Table 5:** Logistic regression of factors associated with at least one prescription of analgesic drugs within one year (n = 9,233)

	**Model 1: OR**_**crude **_**(95% CI)**	**Model 2: OR**_**adj.**_**(95% CI)**	**Model 3: OR**_**adj.**_**(95% CI)**
Dementia group (vs. control group)	1.12 (1.01-1.24)	0.96 (0.86-1.07)	0.78 (0.68-0.88)
Female sex (vs. male)		1.39 (1.28-1.52)	1.16 (1.05-1.28)
Age groups, in years			
65-74		1	1
75-84		1.09 (0.99-1.20)	0.96 (0.86-1.07)
85+		1.10 (0.97-1.24)	1.10 (0.96-1.27)
Level of long term care			
None		1	1
I		1.46 (1.25-1.70)	1.08 (0.91-1.30)
II		1.69 (1.40-2.03)	1.30 (1.05-1.62)
III		1.26 (0.91-1.76)	1.06 (0.73-1.55)
Nursing home residence (vs. community-dwelling)		1.15 (0.94-1.41)	1.11 (0.88-1.40)
Number of prescribed medications			
Q1 (0–4)			1
Q2 (5–7)			2.22 (1.94-2.54)
Q3 (8–11)			3.79 (3.31-4.34)
Q4 (12–51)			7.65 (6.58-8.89)
Cancer pain (vs. no)			1.08 (0.88-1.32)
Pain due to arthritis or osteoarthritis (vs. no)			2.39 (2.17-2.64)
Pain due to fractures (vs. no)			2.86 (2.11-3.88)
Pain due to multimorbidity and care dependency (vs. no)			1.11 (0.89-1.38)
Headache (vs. no)			1.03 (0.83-1.28)
Neuropathic pain (vs. no)			1.24 (1.09-1.41)
Back pain (vs. no)			2.29 (2.07-2.53)
Pain, not elsewhere classified (vs. no)			2.77 (2.21-3.47)

OR = Odds Ratio; CI = Confidence Intervals; Q1, Q2, Q3, Q4 = 25th, 50th, 75th and 100th percentile.

## Discussion

### Findings, comparison with other studies and interpretation

We found several interesting results. Diagnoses indicating pain during the year of observation were highly prevalent with no difference between patients with a first diagnosis of dementia and controls. This was also the case when we adjusted for all other covariates in a multivariate analysis (data not shown). This finding might contradict the widespread perception that pain or conditions associated with it often remain undetected in dementia [5-8]. However, pain management still seems to receive less attention in patients with dementia, which is also suggested by a recent study of nursing home residents [13]. Although the probability of receiving at least one analgesic drug was slightly higher for patients with incident dementia in our crude analysis, the results of the adjusted logistic regression show a significantly lower chance for patients with dementia, when controlled for diagnoses indicating pain and comorbidity. Interestingly, this result still remains significant when only adjusting for one of these measures or when excluding antidementia drugs as well as analgesics from the number of prescribed medications in model 3 (data not shown), underlining the robustness of our findings. Therefore, we can conclude that drug treatment is often not initiated for some dementia cases. We also found that the proportion of patients with diagnoses indicating pain was much higher than the proportion of patients with analgesic prescriptions in dementia (74.4% vs. 47.5%) as well as in controls (72.5% vs. 44.7%). An awareness/recognition problem might be an explanation for this finding. Our results further emphasize that comorbidities should be taken into account when prescription patterns are compared between patients with and without dementia.

In contrast to other studies examining the treatment of dementia patients with analgesics [8-16], we investigated a sample with a first diagnosis of dementia. We rather had expected not to find any differences in analgesic prescriptions between patients with and without dementia in this sample, as communication might not be heavily impaired at this stage. Our findings raise the question if differences in pain medication will become even more apparent with the progression of the disease. This longitudinal perspective should be considered in further research.

Previous efforts to improve the management of pain in patients with dementia focused on recommendations for a better detection of pain by physical examination and the assessment of pain using self-report and observational methods [31,32]. In our study, dementia patients were given less pain medication despite comparable pain related diagnoses. This result suggests that in addition to the difficulties in pain recognition, other factors may influence the decision to start pain medication in patients with dementia. The results of the logistic regression analysis revealed that women, patients with long term care dependency level II, patients with greater comorbidity and pain related to musculoskeletal problems had a greater chance of being prescribed analgesic drugs. Patients with more comorbidities use more medications and receive more often prescriptions of analgesics. A further interpretation might be that pain medication is often prescribed to control pain in the context of mobility. If mobilization is not a primary goal any more, as this is often the case in long term care level III, this can lead to a justified or unjustified reduction of pain medication. Pokela et al. reported a similar association between self-reported mobility and analgesic use in older persons [33]. Studies investigating differences in other medications between patients with and without dementia discussed further explanations for undertreatment: Löppönen et al. found an undermedication for cardiovascular diseases in patients with dementia, especially a less frequent treatment with antithrombotic agents in stroke and beta-blockers in case of hypertension [34]. They forwarded that caution may be an explanation, as older subjects with dementia are known to have an increased risk of falls leading to trauma. In an exploratory German study, Müther et al. found inconclusive results when comparing medications for diabetes, hypertension or hyperlipidemia in patients with dementia compared to controls [35].

There were noticeable differences in the prescribing habits of analgesics between the two groups with liquid oral substances like metamizole and tramadol as well as transdermal opioids more often used in dementia. The latter finding is in line with the study of Bell et al. [18]. Although morphine, oxycodone, or hydromorphone given by the oral route are recommended as first choice step III opioids, transdermal fentanyl and buprenorphine are alternatives for patients with swallowing problems [36], as in some cases of advanced dementia. However, transdermal fentanyl is also often used as a first-line step III opioid in patients without these diagnoses in Germany [37]. The frequent use of liquid oral substances and especially metamizole might also be related to swallowing problems in dementia patients. However, metamizole is associated with potentially life-threatening agranulocytosis [38,39] and caution is indicated, particularly when prescribed over long periods.

### Strengths and limitations

Using claims data for research has many advantages but also disadvantages. On the one hand selection bias is reduced, as claims data allow us to study diagnoses indicating pain and analgesics prescriptions in dementia patients irrespective of their current state of health, communication problems, old age, frailty, institutionalisation or location. Also we could control for further patient related factors such as level of care dependency, and the number of prescribed medications as comorbidity measures.

On the other hand, validity of diagnoses should be considered [40]. Although we studied newly diagnosed patients, we know that the coding of dementia diagnoses is made rather late due to taboo, stigmatization, difficulty in the diagnostic process, and poor therapeutic options [41-43]. Therefore, our sample may partially consist of patients in more advanced stages of dementia. This is also suggested by the fact that 24% of the incident cases were already assigned to care levels II and III. We also did not distinguish between different types of dementia as within our data set more than half of the coded dementia diagnoses are F03, unspecified dementia. This is probably due to the fact that a more precise diagnosis is not needed for reimbursement issues, the primary function of claims data. However, pain identification and management of pain should not be severely impaired by the absence of a precise etiological dementia diagnosis. In Germany, validation studies are scarce [40] and no data on the validity of the dementia diagnosis as well as of the types of pain are available.

It should further be kept in mind that we analyzed diagnoses indicating pain and not pain itself. We do not know if these persons really are in pain and for which indication analgesics were prescribed. Therefore, it was not possible to study the quality of analgesic prescribing and we only used the indicator ‘at least one analgesic’. Furthermore, we do not know whether drugs were prescribed as permanent, acute or as-needed medication, which could be relevant in the case of pain medication [16]. Paracetamol is one of the most used analgesics and in some studies more frequently used in patients with than in those without dementia [15,17]. Unfortunately we could not analyze its use as it is an OTC drug and not documented in claims data. Some of the included step I drugs (ibuprofen, naproxen, diclofenac) are also available as OTC drugs and only reimbursed - and thus included in claims data - at higher doses. For stage II and III analgesics, we are likely to have a fairly good coverage using claims data. Finally, we used data of one health insurance fund only and there are differences between these funds in Germany, for example with respect to age, sex, socioeconomic status and morbidity [44,45]. Thus extrapolations of analyses of single funds to the whole German population should be performed with caution. However, there seems to be no obvious reason for treatment differences in patients with dementia between different health insurance funds.

## Conclusions

Our findings show a comparable documentation of diagnoses indicating pain in persons with incident dementia compared to those without. However, when taking comorbidity into account, there still seems to be an undertreatment of pain in dementia. This might indicate an awareness/recognition problem. Irrespective of dementia, analgesics seem to be more often prescribed to sicker patients and to control pain in the context of mobility. Special attention concerning pain medication should therefore be paid in immobilized patients, as immobilization can lead to a (justified or not) reduction of pain medication. Prescription of analgesics in transdermal or liquids forms may be alternatives in demented patients who suffer from swallowing disorders, but might cause other problems. Our findings raise the question what further reasons might lead to the undertreatment of pain in dementia and what changes occur in the progression of the disease. This longitudinal perspective should be taken up in further research.

## Competing interests

The authors declare that they have no competing interests.

## Authors’ contributions

FH and HK conceptualized the study design and wrote the paper. FH and BW performed the statistical analyses. All authors interpreted the data, critically revised the manuscript, read and approved the final version. All authors read and approved the final manuscript.

## Pre-publication history

The pre-publication history for this paper can be accessed here:

http://www.biomedcentral.com/1471-2318/14/20/prepub
